# Human Infection with *Schineria larvae*

**DOI:** 10.3201/eid1304.061151

**Published:** 2007-04

**Authors:** Max Maurin, Jeanne Noelle Delbano, Léandre Mackaya, Henri Colomb, Christophe Guier, Aziza Mandjee, Christine Recule, Jacques Croize

**Affiliations:** * Centre Hospitalier Universitaire de Grenoble, Grenoble, France; † Hôpitaux Drôme Nord, Romans sur Isere, France

**Keywords:** Schineria, Schineria larvae, bacteremia, humans, myiasis, letter

**To the Editor:** Myiasis remains prevalent worldwide (*1**,**2*) and is infestation by larvae from fly species of live or dead tissues from vertebrate hosts (*1**,**3**,**4*). In humans, myiasis most frequently causes infection of exposed ulcers or traumatic wounds (*1*). In industrialized countries, most cases occur in tourists returning from tropical and subtropical areas (*5**,**6*), but autochthonous cases still exist. Several bacterial species have been associated with fly larvae, including species of the family *Enterobacteriaceae* and, more recently, *Schineria larvae* (*7**,**8*). *S. larvae*, a gram-negative bacterium, has been grown from larvae of *Wohlfahrtia magnifica*, a fly species responsible for myiasis (*7**,**8*). Its 16S rRNA gene has been amplified from a bacterial community of species involved in aerobic thermophilic bioprocesses (*9*). We report a case of *S. larvae* bacteremia in a man with wound myiasis.

On June 12, 2006, a 76-year-old man who had type 2 diabetes mellitus was examined at the emergency department of Drôme North Hospitals, Romans, France, for inflammation of chronic cutaneous ulcers of both legs and intermittent fever. The patient lived alone in a rural, crowded area and had received no medical care. He reported owning sheep and denied any recent travel outside France. At the time of admission, his body temperature was 37.8°C, he was malodorous, and he had swelling and painful wounds on both legs. Maggots were found in the leg wounds, scrotum ulcers, and at the anal margin. A radiographic examination of both legs did not show any osteolytic lesion. Laboratory data were as follows: C-reactive protein 71 mg/L (reference value, <5 mg/L), leukocyte count 18.2 × 10^9^/L (81% granulocytes), platelet and erythrocyte counts within normal limits, glucose 200 mg/dL, hemoglobin A1c level 13.8%. Serum protein electrophoresis showed hypoalbuminemia (20 g/L) and hypergammaglobulinemia (16.9 g/L) but no monoclonal gammopathy. Two blood samples and exudate from the leg wounds were collected for microbial cultures.

The patient was given a combination of amoxicillin-clavulanate and ofloxacin, and his cutaneous wounds were cleaned. After 24 h of incubation, leg wound cultures grew methicillin-susceptible *Staphylococcus aureus*, and the 2 blood cultures yielded the same *S. aureus* strain and an oxidative gram-negative bacterium (Romans strain). The Romans strain was found to be highly susceptible to β-lactams, aminoglycosides, chloramphenicol, cotrimoxazole, fluoroquinolones, and colistin. The antimicrobial drug therapy was changed to oxacillin and ofloxacin. The patient’s condition improved rapidly, and his leg wounds healed progressively during hospitalization. He was discharged 27 days after initiation of antimicrobial therapy, which he continued for 7 more days. The patient was reexamined 1 month later and was considered cured.

The Romans strain was sent to the bacteriology laboratory at Grenoble University Hospital for identification. Using the API 20NE and Vitek II ID-GNB systems (bioMérieux, Marcy L’Etoile, France), we obtained, respectively, a “good” identification of *Psychrobacter phenylpyruvicus* and a “very good” identification of *Oligella ureolytica*. The nearly complete 16S rRNA gene of the Romans strain was amplified and sequenced; primers were Fd1 and rp2 (*10*) (GenBank accession no. EF120377). A BLAST search that used the network service of the National Center for Biotechnology Information (www.ncbi.nlm.nih.gov/) showed 99.6% identity between the determined gene sequence of 16S rRNA and that of *S. larvae* type strain L1/68^T^ (accession no. AJ252143). The 16S rRNA gene sequences from several species belonging to the Gamma Proteobacteria order were aligned by using the ClustalW package (www.ncbi.nlm.nih.gov/). A consensus phylogenetic tree was constructed from Jukes-Cantor evolutionary distances based on the neighbor-joining method using *Bacillus subtilis* as the root. The Romans strain clustered with previously characterized *S. larvae* strains (Figure).

**Figure:**
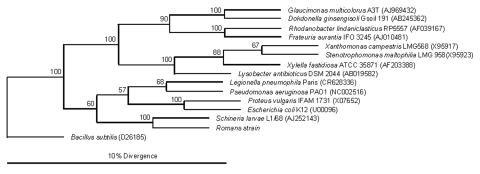
Phylogenetic position of the Romans strain within the Gamma Proteobacteria, determined by using Jukes-Cantor evolutionary distance calculation and neighbor-joining tree method. Bootstrap values (based on 500 steps) are indicated. GenBank accession no. of 16S rRNA gene of each bacterial species is indicated in parentheses.

Our report demonstrates that *S. larvae* can induce bacteremia in humans. Because *S. larvae* has been associated with only fly larvae, we can speculate that bacteremia originated from maggots infesting the patient’s wounds. We cannot affirm that *W. magnifica* was the fly species involved because maggots were not saved for identification. Phenotypic identification of *S. larvae* is tedious (*7*). Because it is an asaccharolytic species, erroneous identification may occur. We can speculate that difficulties in phenotypic identification of this species may explain why it has not been previously reported as a potential human or animal pathogen.

In conclusion, myiasis remains an unresolved problem in animals and humans worldwide. Physicians and microbiologists should be aware of the possibility of *S. larvae* bacteremia and should specifically search for *S. larvae* infection in myiasis patients. Also, animal myiasis is still responsible for severe economic losses to the livestock industry worldwide. The occurrence of *S. larvae* bacteremia in animals with myiasis may explain the evolution from disease to death, especially in chronically infected animals.
